# Reticulate acropigmentation of Kitamura with café-au-lait macules: a rare case report

**DOI:** 10.3389/fmed.2025.1647451

**Published:** 2025-08-29

**Authors:** Bukuan Gao, Huijuan Wang, Qianqian Fan, Lin Cong, Guoqiang Zhang, Xiaoye Liu

**Affiliations:** ^1^Department of Cosmetic Dermatology, Beijing Yixing Medical Cosmetology Hospital, Beijing, China; ^2^Department of Dermatology, The First Hospital of Hebei Medical University, Shijiazhuang, China; ^3^Department of Cosmetic Dermatology, Shenzhen Yixing Medical Cosmetology Hospital, Shenzhen, China

**Keywords:** reticulate acropigmentation of Kitamura, café-au-lait macules, coexistence, rare case, treatment

## Abstract

Reticulate acropigmentation of Kitamura (RAK) predominantly affects East Asian populations, though isolated cases and familial occurrences have been reported globally. Japanese researchers Kitamura et al. first described this condition in 1943. In 2013, pathogenic variants in ADAM10 (a disintegrin and metalloprotease 10) were identified as causative in multiple Japanese RAK pedigrees. The occurrence of RAK with Dowling-Degos disease (DDD) is relatively well-documented. However, rare associations with bilateral clinodactyly, nevus of Ito, dermatopathia pigmentosa reticularis, and progressive seborrheic keratosis have also been reported. RAK is an extremely rare autosomal dominant disorder. Café-au-lait macules (CALMs) represent common hyperpigmented lesions, yet no documented cases of RAK-CALMs coexistence exist in the literature to date.

## Introduction

Reticulate acropigmentation of Kitamura (RAK) is an exceedingly rare autosomal dominant disorder. First described by Japanese researchers Kitamura et al. in 1943 (1), its pathogenesis was later linked to *ADAM10* (a disintegrin and metalloprotease 10) mutations in multiple Japanese pedigrees in 2013 (1). Characteristic manifestations include reticulate or punctate, hyperpigmented, slightly depressed macules on acral sites without hypopigmentation. Some patients exhibit pigment extension to the neck and axillae, with rare involvement of the trunk and face. However, RAK-associated freckle-like, pinhead-sized hyperpigmented macules have been documented on the bilateral upper eyelids and cheeks (2). Most cases present in early childhood with progressive intensification. Beyond classic pigmentation, punctate atrophy and palmoplantar keratoderma may occur. The precise prevalence remains undetermined, though global estimates suggest fewer than 1 per 1,000,000 individuals (3). Café-au-lait macules (CALMs) represent hypermelanotic lesions that may affect any cutaneous surface exceptthe palms and soles. The coexistence of RAK and CALMs constitutes a rare clinical phenomenon. We describe a novel case of concomitant RAK and CALMs, analyze their potential correlation, and expand the spectrum of hyperpigmentary disorders.

**Figure 1:**
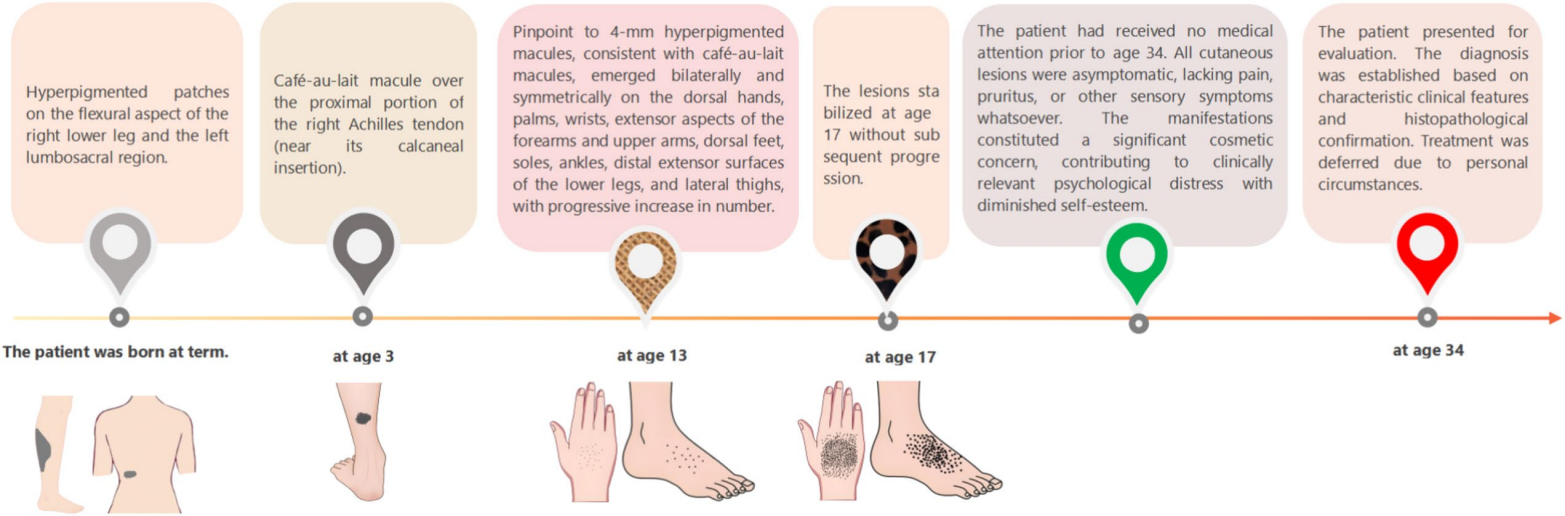
Flow chart of disease progression.

A 34-year-old Han Chinese woman presented with café-au-lait macules on the right calf flexural region and left lumbosacral area that were noted at birth, and exhibited proportionate growth. A parent reported that similar lesions had developed over the right Achilles tendon at age 3 years, and enlarged proportionally. She presented with progressive acral hyperpigmentation that had evolved over the past two decades. The patient initially developed asymptomatic punctate pigmented macules on her hands and feet without identifiable triggers that gradually extended to involve the wrists, extensor aspects of the forearms, upper arms, ankles, distal lower extremities, and lateral thighs. The patient’s general health remained unaffected, with a normal systemic physical examination and no significant med ical or family history. The patient is non-consanguineous and nulliparous, with no similar dermatological conditions reported in her family (Figure 1).

**Figure 2:**
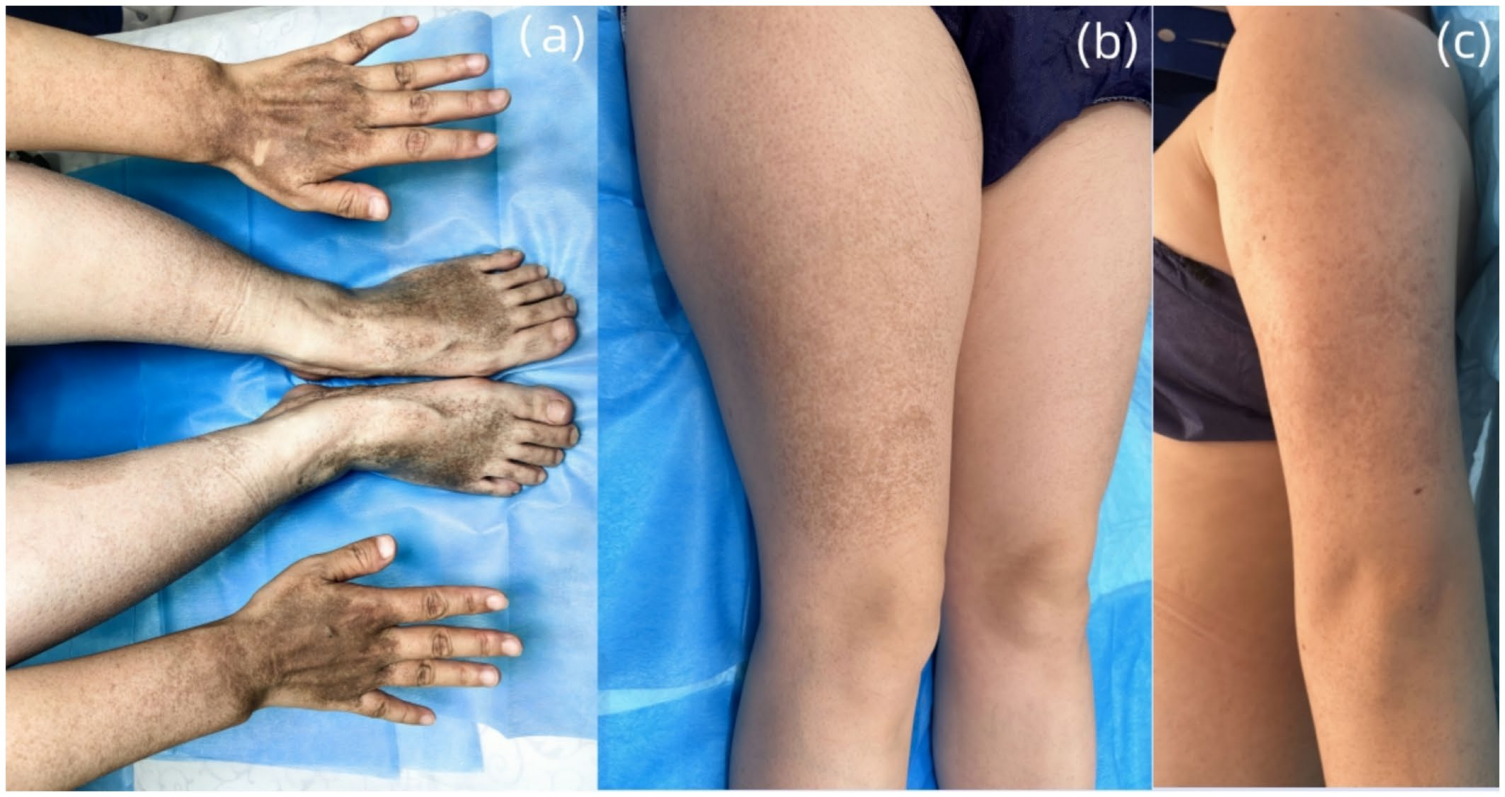
**(A)** Multiple pinhead-to mung bean-sized brown macules are present on the dorsal hands and feet. **(B,C)** Brown macules, 2 to 6 mm in diameter, were observed in reticulated clusters on the extensor surfaces of the thighs and lateral aspects of the upper limbs.

**Figure 3:**
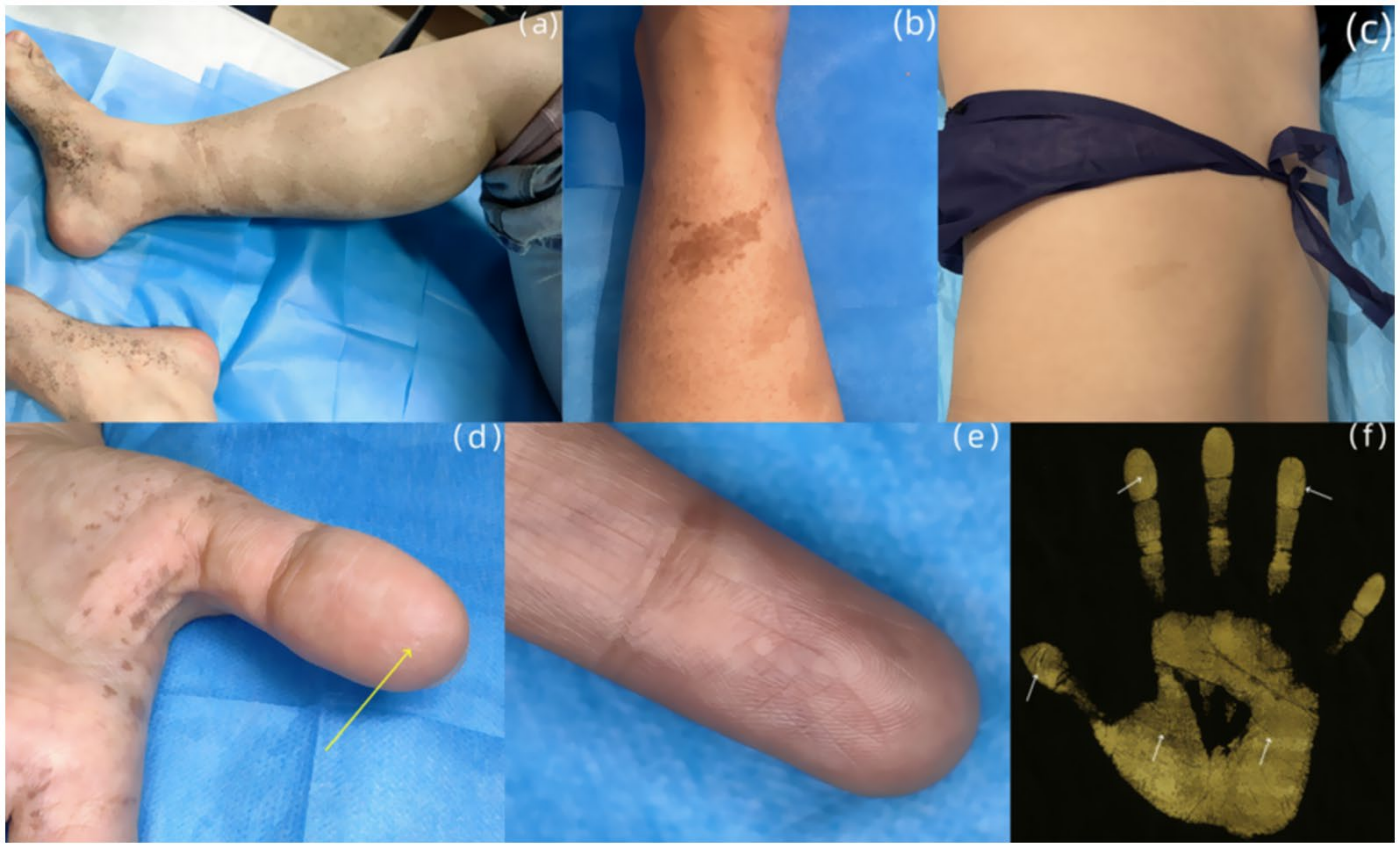
**(A)** An irregularly shaped, well-demarcated light café-au-lait patch is seen on the posterior aspect of the right calf. **(B)** A 5 × 2-cm café-au-lait patch was observed superior to the right Achilles tendon. **(C)** An irregularly shaped, pale café-au-lait patch with well-defined borders was noted on the left lumbosacral region. **(D)** Punctate pitting (yellow arrow) is observed on the digital pulp of the left thumb. **(E)** Multiple interruptions of the dermatoglyphics is seen on the left index finger. **(F)** Dermatoglyphic examination: Partial breaks in palmar and digital ridges were detected on both hands (indicated by white arrows).

Dermatological examination revealed scattered pinhead-to-mung bean-sized brown macules distributed bilaterally on the dorsal hands, palms, wrists, extensor forearms, upper arms, dorsal feet, plantar surfaces, ankles, distal lower extremities, and lateral thighs. Partial lesions coalesced into reticulated patterns with unaffected intervening skin, accompanied by mild cutaneous depressions in some hyperpigmented areas was observed (Figures 2A–C). A well-demarcated 20 × 10 cm café-au-lait patch was present on the right posterior calf (Figure 3A). A 5 × 2-cm café-au-lait patch was observed superior to the right Achilles tendon, with scattered mung bean-sized café-au-lait macules at its periphery (Figure 3B). A 5.0 × 1.5-cm irregularly shaped, pale café-au-lait patch with well-defined borders was noted on the left lumbosacral region (Figure 3C). Punctate pitting was observed on the digital pulps and partial interruption of palmar creases/dermatoglyphics (Figures 3D–F). There was no facial, cervical, truncal, or mucosal involvement. Palmoplantar hyperkeratosis was absent. The patient had normal hair and nail morphology.

**Figure 4:**
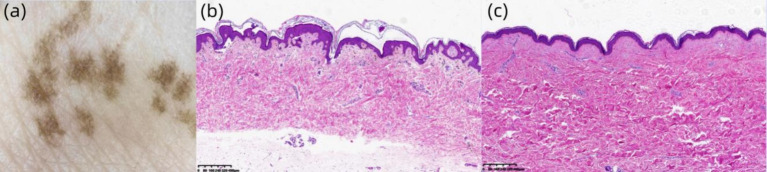
**(A)** Under polarized light, multiple hyperpigmented structures with irregular distribution are observed (original magnification ×30). **(B)** Hematoxylin and eosin (H&E) staining (×40) demonstrates mild hyperkeratosis in the epidermis, increased pigment granules in the basal layer, and scattered pigment incontinence within the dermis. **(C)** H&E staining (×40) reveals mild epidermal hyperkeratosis, irregular acanthosis in the stratum spinosum, basal layer hyperpigmentation, and focal pigment incontinence in the dermis.

Laboratory findings: Routine blood, urine, and stool examinations revealed no abnormalities. Dermoscopic examination (Left Dorsal Hand): Under polarized light, multiple irregularly distributed hyperpigmented structures were observed interspersed with areas of normal skin (Figure 4A). Histopathological analysis of the right dorsal foot (RAK lesion): The epidermis showed mild hyperkeratosis with increased pigment granules in the basal layer. The dermis exhibited scattered pigment incontinence, mucoid material deposition between the collagen fibers, and mild perivascular lymphocytic infiltration (Figure 4B). Histopathological analysis of the right calf flexor region (CALS lesion): The epidermis demonstrated mild hyperkeratosis and irregular acanthosis along with basal layer hyperpigmentation. The dermis revealed scattered pigment incontinence, mucoid material deposition between the collagen fibers, and mild perivascular lymphocytic infiltration (Figure 4C).

Due to childbearing considerations, the patient currently declines intervention for RAK and CALS.

## Discussion

This report describes the first documented co-occurrence of RAK and CALMs. While associations between RAK-Dowling-Degos disease (DDD) are well-established (4), and rare cases link RAK with bilateral clinodactyly, nevus of Ito, dermatopathia pigmentosa reticularis, and progressive seborrheic keratosis (3, 5–7), no RAK-CALMs co-occurrence has been reported to date. Although RAK and CALMs have distinct genetic origins, their coexistence in a single individual compounds both esthetic concerns and therapeutic challenges.

*ADAM10* located at 15q22, comprises 16 exons. Its protein product cleaves the extracellular domain of Notch receptors, activating Notch signaling and disrupting melanin transport. Currently, only 15 pathogenic *ADAM10* variants are catalogedin the Human Gene Mutation Database (HGMD), distributed across exons and introns without mutational hotspots (8).

Dermatoglyphic breaks and pitted depressions are characteristic of RAK. Koguchi et al. demonstrated enhanced visualization of palmar pits using India ink imprinting (9). Our patient exhibited classic RAK features: fingerprinting with blue ink on A4 paper—scanned and inverted—revealed bilateral palmar and digital ridge discontinuities. Dermoscopy showed hypopigmented circular pits on finger pads. Notably, not all RAK cases present with palmar pits or ridge disruptions (10); Indian studies report dermoscopic pigmentation within these pits (11, 12), suggesting phenotypic heterogeneity across ethnicities (13).

RAK requires differential diagnosis from dyschromatosis symmetrica hereditaria (DSH) and DDD. DSH is characterized by the simultaneous presence of hyperpigmented and hypopigmented macules in the acral regions, and ADAR1 has been identified as the causative gene. Some DDD patients may exhibit clinical features overlapping with RAK; however, pigmentation in DDD typically demonstrates a more extensive distribution, predominantly affecting the flexural areas, and is often accompanied by punctate keratotic lesions. Pathogenic genes associated with DDD include KRT5, POFUT1, and others.

CALMs affect 0.3–27% of individuals depending on age/ethnicity (14). These well-demarcated, light-to-dark brown macules (0.1–20 cm) may appear anywhere except palms/soles. Segmental CALMs occur in mosaic patterns. While typically benign, multiple CALMs signal syndromes like neurofibromatosis or McCune-Albright.

Effective therapeutic strategies for RAK are currently being investigated. Several studies have reported that topical agents, including retinoids, azelaic acid, and corticosteroids, demonstrate potential efficacy in managing RAK (15, 16). Additionally, treatment with 532-nm and 755-nm Q-switched lasers has been associated with an improved appearance of skin lesions in documented cases (17, 18). However, these findings are predominantly derived from isolated case reports. Currently, the primary treatment for CALMs for 532-nm and 755-nm lasers with nanosecond or picosecond pulse durations (19–21). Although substantial research on laser applications for benign pigmented dermatoses has been reported, no standardized protocols have been established for specific treatment parameters or combination regimens. The 2022 German Society of Dermatology guidelines on cutaneous laser therapy designate laser treatment as a first- and second-line therapeutic option for hyperpigmentary disorders (22).

## Conclusion

Current evidence indicates that RAK and CALMs represent distinct pigmentary disorders arising from separate genetic mechanisms, thus their coexistence in a single individual constitutes a rare coincidence. Due to the rarity of RAK, large-scale data are lacking. Although the limited available case reports indicate some treatment efficacy resulting in lesion fading, outcomes are often suboptimal and fail to achieve complete or satisfactory clearance. Furthermore, longitudinal data on long-term efficacy are notably absent. No treatment currently exists that completely removes CALMs lesions. Traditional pharmacologic therapies carry risks of adverse effects, whereas laser therapy offers superior efficacy and safety, and has emerged as the current mainstream treatment. The overall response rate for laser treatment of CALMs ranges from 20.0 to 74.4% (23), but recurrence rates are high (5–50%), and recurrence is unpredictable in nature (24). Clinically, the concurrent presentation of these two conditions significantly increases the inherent therapeutic challenge. Existing literature indicates that RAK can co-occur with various other hyperpigmentary disorders, underscoring the complexity and diversity within this disease spectrum. From the patient perspective, beyond cosmetic concerns, the extreme rarity of this unique presentation exacerbates feelings of isolation. The current case is a valuable reference for clinicians and patients with similar presentations, with respect to medical and psychosocial implications.

## Data Availability

The original contributions presented in the study are included in the article/supplementary material, further inquiries can be directed to the corresponding author/s.
